# Relevant Predictors in the Association Between Patients’ Functional Status and Scar Outcomes After Total Hip Arthroplasty

**DOI:** 10.7759/cureus.50702

**Published:** 2023-12-17

**Authors:** Madalin Bulzan, Simona Cavalu, Florica Voita-Mekeres

**Affiliations:** 1 Orthopedics and Traumatology, Faculty of Medicine and Pharmacy, University of Oradea, Oradea, ROU; 2 Therapeutics, Faculty of Medicine and Pharmacy, University of Oradea, Oradea, ROU; 3 Morphological Disciplines, University of Oradea, Oradea, ROU

**Keywords:** traumatic injury, coxarthrosis, scar acceptance, adl (activities of daily living), total hip arthroplasty (tha)

## Abstract

Background: We aimed to investigate the relevant predictors in the association between the functional status and the consequences of the persistence of scars in patients with traumatic versus non-traumatic coxarthrosis after total hip arthroplasty (THA).

Methods: A total of 203 patients undergoing THA after traumatic or non-traumatic coxarthrosis were asked to complete the Mekeres' Psychosocial Internalization Scale (MPIS), in which they self-evaluated on a Likert scale (between one and five) by selecting the rating that corresponded to their personal opinion and the activities of daily living (ADL) form at six months postoperative. The statistical data were processed using the IBM SPSS Statistics software version 22.0 (IBM Corp., Armonk, NY). A combined assessment of the internalization of scars using MPIS and ADL forms after THA allowed for the identification of relevant predictors of the quality of life six months post-surgery in patients with traumatic or non-traumatic coxarthrosis.

Results: Depending on the coxarthrosis etiology (traumatic or non-traumatic), the results were further processed by a univariate ANOVA, considering the independent variables represented by symptoms, the number of surgical procedures, and the postoperative evolution, which are acting on the outcomes of physical functioning (the dependent variable) in the postoperative phase. In the case of the traumatic group, our results suggest that the number of surgical interventions, the ability to internalize scars, and autonomy in terms of body care are predictors of the quality of life. In patients with non-traumatic coxarthrosis, an important role in predicting quality of life is played by the administered treatment and the ability to maintain their autonomy regarding self-hygiene six months post-surgery.

Conclusions: The predictive regression equation suggests that the quality of life in patients with traumatic coxarthrosis can be predicted by the number of surgical interventions, the administered treatment, the ability to internalize scars, and the autonomy regarding body care activities. On the other hand, for patients with non-traumatic coxarthrosis, an important role in predicting the quality of life is played by the treatment and the ability to maintain autonomy in terms of body hygiene activities.

## Introduction

Coxarthrosis, or osteoarthritis (OA), of the hip joint, is one of the most serious orthopedic diseases because, as perceived worldwide, it significantly affects the quality of patients’ lives. Osteoarthritis is characterized by the slow, irreversible breakdown of articular cartilage, particularly in the hip or knee [1-3]. It affects the joint in terms of its mechanical functioning and gradually leads to its complete destruction. Osteoarthritis is a chronic condition manifested by pain, stiffness, and swelling, with long-term impairment of normal daily physical activities. In cases of advanced OA, the most effective method of treatment remains surgical intervention [4-6].

Total hip arthroplasty (THA) is the standard method for treating OA; it is considered the most successful orthopedic surgery, resulting in a major improvement in the quality of life by eliminating the pain and regaining joint mobility [7, 8]. Arthroplasty techniques have evolved rapidly in recent years in the context of the development of new biomaterials and orthopedic implants, with or without cementation [9]. Currently, hip replacement is done through minimally invasive surgery. The incisions have reduced in size, the muscle injuries are minimal, and the patient is mobilized 24 hours after the intervention [10, 11].

Effective healing of skin lesions is crucial to ensuring skin barrier function, but pathological wound healing and scar formation can affect the patient both physiologically and psychologically [12-14]. Scars represent long-term physical signs, and if they were produced after hip arthroplasty, patients must accept their presence on the hip. Although many articles have been published regarding the management of hypertrophic and keloid scars, there are no studies evaluating the psychosocial effects of post-hip arthroplasty scars [15]. Mekeres' Psychosocial Internalization Scale (MPIS) is a scale validated in 2021 that evaluates the psychosocial effects of posttraumatic scars [16].

The term "activities of daily living (ADL)" was first used by Sidney Katz (1950) to refer to the basic skills that a person needs in ordinary everyday life (body hygiene, dressing, going to the toilet, sphincter continence, and eating) [17, 18]. As ADL serves as an indicator of a person's functional status, it is often related to other terms such as functional ability or functional impairment. The ADLs essentially indicate their ability to self-care without assistance [19, 20].

In this study, we aimed to investigate the relevant predictors in the association between functional status and the consequences of the persistence of scars in patients with traumatic versus non-traumatic coxarthrosis after THA.

## Materials and methods

This prospective cross-sectional study was carried out between October 1, 2020, and September 1, 2022, in the Emergency County Clinical Hospital, Oradea, Romania, in the orthopedics department. The study included a total of 203 participants and was approved by the Institutional Review Board and Ethical Council of the Emergency County Clinical Hospital, Oradea, Romania (approval numbers: 1267/14.01.2022 and 1087/13.01.2022). The research was conducted in compliance with the Declaration of the World Medical Association of Helsinki. Participation in the study was voluntary, and written informed consent was obtained from all participants for the accurate collection of information and data processing.

Inclusion and exclusion criteria were as follows: adults who underwent THA were included in the study. Smokers, patients with a recent history of SARS-CoV-2, patients with multiple comorbidities (including malignant tumors or organ failure), patients who refused to participate in the study, and patients who refused to sign the informed consent were excluded.

Respondents were asked to complete the MPIS, in which they self-evaluated on a Likert scale (between one and five) by selecting the rating that corresponded to their personal opinion (Appendices A-B), as well as the ADL form six months postoperatively, to assess the psychosocial effects of the scars and the functional state of the hip. The collected data were processed using the statistical program IBM SPSS software version 22.0 (IBM Corp., Armonk, NY).

Depending on the coxarthrosis etiology (traumatic or non-traumatic), the results were analyzed by a univariate ANOVA, considering the independent variables represented by symptoms, the number of surgical procedures, and the postoperative evolution, which are acting on the outcomes of physical functioning (the dependent variable) in the postoperative phase.

At the predictive level, depending on the etiology of coxarthrosis, we consider that the internalization of scars (measured with MPIS), the number of surgical interventions, THA, and ADL (body hygiene) are hypothetical predictors of the quality of life postoperatively. For this purpose, the data were processed using the predictive regression equation.

Tools used for the study

1. The MPIS (Appendix A) is a scale that evaluates the psychosocial effects produced by post-traumatic scars. Each study participant answered 15 questions and scored from one (disagree) to five (strongly agree) using the Likert scale. The total score of the MPIS is between 15 and 75. Moreover, it proved to have good internal consistency (Cronbach, 0.943), with the scale highlighting the patient's concerns regarding the scar, the morphological aspect of the scar, the gender, as well as the professional and social impact of affected patients [16, 20, 21].

2. The ADL form (Appendix B) is a tool that quantifies a person's ability to perform everyday tasks to determine their level of autonomy. Six basic needs in the ADL (locomotion, continence, eating, hygiene, toileting, and dressing) are quantified between 0 (dependent) and two (autonomous), the total score being from 0 to 10, which corresponds to four stages: stage I being autonomy (10 points), stage II being semi-independent (eight to 10 points), stage III being assisted independence (three to eight points), and stage IV being total dependence (0 to three points) [22,23].

## Results

A total of 203 participants were enrolled in this study, aged between 24 and 90 years (m = 58.44; AS = 17.41), of whom 92 were women, representing a percentage of 45.3%, and 111 were men, representing a percentage of 54.7%. We analyzed the predictors that can hypothetically play an important role in patients’ quality of life, considering the etiology of coxarthrosis (traumatic or non-traumatic). The preliminary analysis phase supported compliance with the conditions regarding homogeneity and multicollinearity. Demographic information of the patients is presented in Table 1.

**Table 1:** Demographic information of the patients included in the study

Variable		Traumatic N=90	Non-traumatic N= 113
Gender	Male	47	64
	Female	43	49
Age	Minimum	27	24
	Maximum	90	89
Year of intervention	2020	24	42
	2021	50	51
	2022	16	20
Diagnostic details	Left coxarthrosis operated	34	14
	Right coxarthrosis operated	33	21
	Unilaterally operated coxarthrosis	21	36
	Bilaterally operated coxarthrosis	2	41
Number of interventions	1	71	49
	2	15	28
	3	4	36
Post-surgery evolution	Favorable	78	58
	Unfavorable	12	55

In Table 2, the inter-correlation matrix of the variables considered predictors of the quality of life six months after surgery is presented.

**Table 2:** Correlations between quality of life and relevant clinical variables in coxarthrosis MPIS: Mekeres’ Psychosocial Internalization Scale; THA: total hip arthroplasty; ADL: activities of daily living; *p<.05; **p<.01; ***p<.001; ns: not significant.

Etiology	Variables	Postoperative quality of life	MPIS	Number of interventions	Treatment
Traumatic N=90	Postoperative quality of life	-			
	MPIS total score	-.408^***^	-		
	Number of interventions	.460^***^	-.202^*^	-	
	THA	.486^***^	-.147 ns	.287^***^	-
	ADL-body hygiene	-.561^***^	.377^***^	-.497^***^	-.431^***^
Non-traumatic N=113	Postoperative quality of life	-			
	MPIS total score	-.090 ns	-		
	Number of interventions	.300^***^	-.340^***^	-	
	THA	.403^***^	-.089 ns	.335^***^	-
	ADL-body hygiene	-.415^***^	.243^***^	-.378^***^	-.364^***^

Quality of life shows direct associations with the number of surgical interventions (r=.460; p<.001) and THA (r=.486; p<.001), while an inverse relationship was noticed with ADL- body hygiene (r=-.561; p<.001) and internalization of scars (r=.408; p<.001) in the case of traumatic coxarthrosis, suggesting the distancing of the two variables. Postoperative quality of life in patients diagnosed with non-traumatic coxarthrosis is associated with the number of previous surgeries (r=.300; p<.001) and with THA (r=.403; p<.001). Similarly, the post-surgical quality of life was dissociated from ADL- body hygiene.

We suggest that the internalization of scars (measured with the MPIS), the number of surgical interventions, THA, and ADL-body hygiene are relevant predictors in estimating the quality of life at six months after surgical intervention in traumatic or non-traumatic coxarthrosis.

Table 3 shows the statistical differences that resulted after processing the data (multilinear regression equation) according to the postoperative quality of life (F(89) = 18.644; p<.001) in participants with traumatic and non-traumatic coxarthrosis (F(112) = 9.285; p<.001).

**Table 3:** Factors influencing the predictive multilinear regression equation, depending on the quality of life after surgery a. Dependent variable: postoperative quality of life b. Predictors: (Constant), activities of daily living (body hygiene), Mekeres' Psychosocial Internalization Scale, total hip arthroplasty, and number of interventions

Etiology	Model	∑ R^2^	Df	R^2^	F	p
Traumatic	Regression	1374.180	4	343.545	18.644	.001^b^
	Residual	1566.247	85	18.426		
	Total	2940.428	89			
Non-traumatic	Regression	592.697	4	148.174	9.285	.001^b^
	Residual	1723.539	108	15.959		
	Total	2316.236	112			

**Figure 1:**
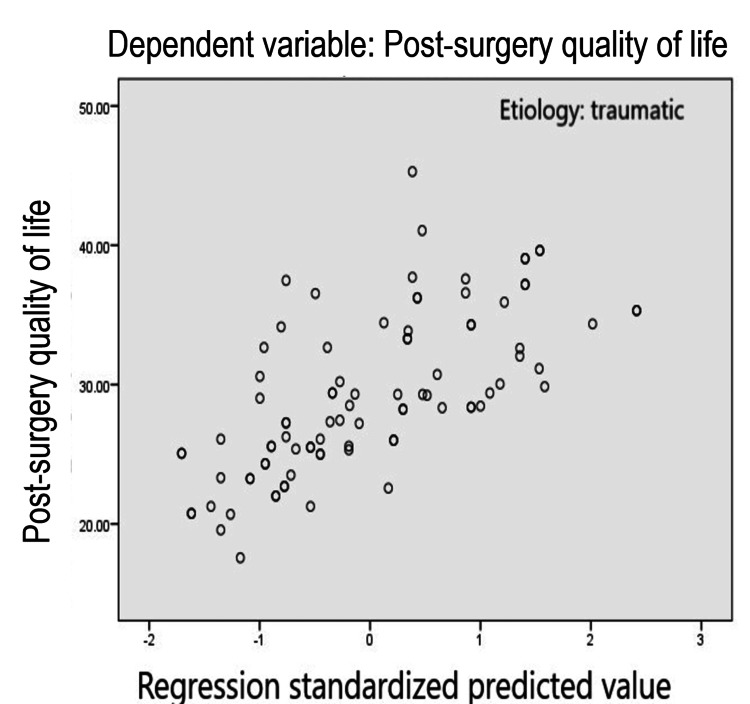
Multilinear regression plot regarding post-surgery quality of life in participants with traumatic coxarthrosis Etiology: traumatic

The multiple determination coefficient (the percentage of the dispersion of the postoperative quality of life) explained by the joint action of the previously mentioned predictors is R^2^=.467, which indicates that the predictors contribute 46.7% to the dispersion of the postoperative quality of life in people diagnosed with traumatic coxarthrosis. This percentage is highly significant and should draw the attention of specialists, as these predictors, in association with other clinically and socially relevant elements, might provide valuable information for healthcare sectors (Table 4, Figure 1).

**Table 4:** The summary model of the predictive multilinear regression equation in the case of participants with different coxarthrosis etiologies, depending on the quality of life after surgery a. Predictors: (Constant), activities of daily living-body hygiene, Mekeres' Psychosocial Internalization Scale, treatment, and number of interventions b. Dependent variable: postoperative quality of life

Etiology	Model	R	R^2^	R^2^ adjusted	Estimated standard error
Traumatic	1	.684^a^	.467	.442	4.29260
Non-traumatic	1	.506^a^	.256	.228	3.99483

**Figure 2:**
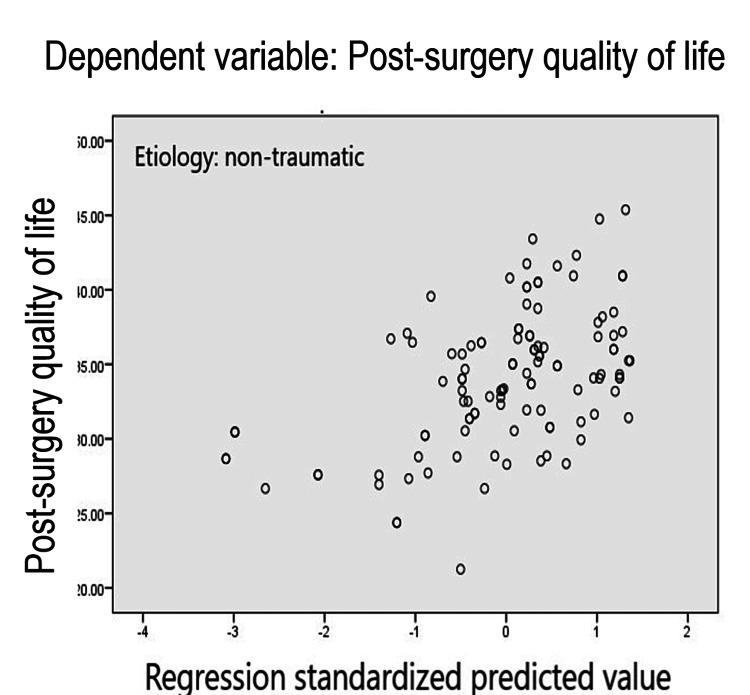
Multilinear regression plot regarding post-surgery quality of life in participants with non-traumatic coxarthrosis Etiology: non-traumatic

On the other hand, the coefficient of multiple determination in participants with non-traumatic coxarthrosis is R^2^=.256, indicating that the predictors contribute 25.6% to the dispersion of quality of life; therefore, there is a possibility that other factors might play an important predictive role (Table 4, Figure 2).

In Table 4, the t-test of significance shows a statistically significant contribution of the predictors to the assessment of the quality of life in traumatic patients, while a lesser extent was noticed in non-traumatic ones.

The number of surgical interventions (β=.208; t=2.275; p<.02) and THA (β=.285; t=3.235; p<.002) highlights a positive relationship with quality of life and also significant β values. Based on the recorded coefficients, we suggest that the predictors are relevant not only for the postoperative quality of life but also for the subsequent recovery, from a medical and psychosocial perspective. The predictors internalization of scars (β=-.231; t=-2.699; p<.008) and ADL-body hygiene (β=-.248; t=-2.417; p<.01) show a negative relationship with the quality of life six months after surgery, which suggests a difficult integration of scar internalization, along with an increased attention to self-care capacity, where the patients' expectations were probably unrealistic.

According to Table 5, the standardized coefficients (β) in the case of traumatic coxarthrosis suggest that a decreased quality of life in terms of personal care and autonomy might generate a high potential for vulnerability to depression, associated with a lower level of realism regarding the aspect of the scars as evidence of the trauma.

**Table 5:** Coefficients of the factors involved in the predictive multilinear regression equation on the post-surgery quality of life THA: total hip arthroplasty; ADL: activities of daily living

Etiology	Model	Unstandardized coefficient		Standardized coefficient	T	P
		β	Error	β		
Traumatic	(Constant)	34.063	3.661		9.305	.001
	Internalization of scars	-.174	.064	-.231	-2.699	.008
	Number of interventions	2.255	.991	.208	2.275	.02
	THA	.885	.274	.285	3.235	.002
	ADL – body hygiene	-2.234	.924	-.248	-2.417	.01
Non-traumatic	(Constant)	28.001	3.583		7.815	.00
	Internalization of scars	.038	.079	.042	.476	.635
	Number of interventions	.622	.506	.118	1.230	.221
	THA	.978	.341	.264	2.873	.005
	ADL – body hygiene	-1.880	.622	-.285	-3.024	.003

The standardized coefficients and the t-test of significance suggest a partial contribution of the predictors to the estimation of the patient's quality of life in the case of non-traumatic coxarthrosis.

Total hip arthroplasty is the most powerful predictor (β=.264; t=2.873; p<.005) in relation to the quality of life in the case of traumatic coxarthrosis, as it presents a positive β value, while the ADL predictor (body hygiene) has a negative impact on the quality of life (β=-.285; t=-3.024; p<.003). Other predictors, such as the number of surgical interventions and the internalization of scars, are not relevant (not predictive) for the subsequent evolution of the quality of life in the case of non-traumatic coxarthrosis.

## Discussion

In this study, we analyzed the relevant predictors for the functional status and psychosocial impact of scars in patients with traumatic or non-traumatic coxarthrosis. The obtained results indicated a percentage of 46.7% of the quality of life in traumatic coxarthrosis being determined by the number of surgical interventions, THA, scar internalization, and body hygiene.

In the case of non-traumatic coxarthrosis, the identified predictors contributed 25.6% to the increase in the quality of life, which indicates that other factors play an important role. However, complex treatment as well as body hygiene or autonomy are useful indicators that need to be followed in the post-surgical stage.

We know from our experience that in the case of coxarthrosis operated unilaterally or bilaterally, the need for partial help for bodily hygiene, or even dependence on another person, increases. The ability to go to the toilet alone has a great psychological impact. As presented in the literature, there is a frequent need for help going to the toilet in patients who underwent THA with an uncemented total prosthesis [24, 25].

Ozawa and Shimizu (2007) showed that the suffering caused by coxarthrosis has a particular impact on the patient’s quality of life, both physically and emotionally. A special role in the assessment of patients' quality of life is played by their emotional state and their acceptance of their functional status [26-28]. Silişteanu and Szakács [26] emphasized the role of quality of life in the case of patients with coxarthrosis and chronic osteoarticular degenerative diseases that represent a public health problem due to the duration of the disease and its family, social, economic, and medical implications.

Quality of life showed direct associations with the number of interventions and administered treatment, while an inverse relationship was noticed with ADL-body hygiene and internalization of scars, suggesting the distancing of the two variables in the case of traumatic coxarthrosis. Postoperative quality of life in patients with non-traumatic coxarthrosis is associated with the number of previous surgeries and surgical treatments. Similarly, in the case of non-traumatic patients, post-surgical quality of life is dissociated from ADL-body hygiene.

Summarizing the results from the predictive regression equation, in the case of traumatic coxarthrosis, we suggest that the quality of life can be predicted by the number of surgical interventions and THA, but also by the ability to internalize scars and by autonomy regarding body care, evaluated six months post-surgery. In the case of patients with non-traumatic coxarthrosis, an important role is played by the administered treatment and the ability to maintain autonomy regarding bodily hygiene.

In orthopedics, the assessment of patients' quality of life in the preoperative and postoperative phases is mentioned with a low frequency compared to other branches of medicine. Our interest was focused on the evaluation of ADL and quality of life in patients with traumatic and non-traumatic coxarthrosis by longitudinal follow-up. The literature indicates that there are no studies that have adequately investigated this issue of major importance in terms of patient recovery. The ADLs are essential routine tasks that most healthy people can perform without assistance. The inability to perform essential activities of daily living can lead to unsafe conditions and a decrease in quality of life. The postoperative care team should be aware of the importance of ADL assessment to ensure proper assistance for vulnerable patients.

The limitations of our study are the unicentric study and prospective design, which did not allow the comparison of the functional states of patients in the pre-and postoperative phases. As a future research project, our intention is to conduct a multi-center study comprising a larger number of patients and a complex analysis including the influence of age and gender associated with functional status and scar outcomes after THA.

## Conclusions

The internalization of scars, number of surgical interventions, treatment, and ADL-bodily hygiene are relevant predictors in estimating the quality of life six months after THA in patients with traumatic or non-traumatic coxarthrosis. The predictive regression equation suggests that the quality of life of patients with traumatic coxarthrosis can be predicted by the number of surgical interventions, the administered treatment, the ability to internalize scars, and the autonomy regarding body care activities. On the other hand, for patients with non-traumatic coxarthrosis, an important role in predicting the quality of life is played by the treatment and the ability to maintain autonomy in terms of body hygiene activities.

## Appendices

Appendix A 

**Table 6:** Mekeres' Psychosocial Internalization Scale (MPIS) Interpretation of the MPIS score: <35 points = psychosocial internalization of the scar; between 35 and 54 points = aesthetic damage; ≥55 points = disfigurement [20].

No.	Item	Score				
1	How attractive did you consider yourself before the occurrence of the scar?	1	2	3	4	5
2	How attractive do you feel after the emergence of the scar?	1	2	3	4	5
3	Are you aware of the presence of the scar?	1	2	3	4	5
4	How much has the presence of the scar changed your life?	1	2	3	4	5
5	Was your relationship with other people negatively affected by the presence of the scar/has it influenced your relationship with others?	1	2	3	4	5
6	Did you need psychological/psychiatric help before the occurrence of the scar?	1	2	3	4	5
7	Did you need psychological/psychiatric help after the emergence of the scar?	1	2	3	4	5
8	Have you ever felt anxious before the scar occurred?	1	2	3	4	5
9	Did you ever feel anxious after the scar was produced?	1	2	3	4	5
10	How has the scar changed your way of interacting with others?	1	2	3	4	5
11	Does the presence of the scar have an impact on your sexual behavior?	1	2	3	4	5
12	Do you think you have a lesser chance of a social or close relationship?	1	2	3	4	5
13	Do you think the presence of the scar reduces your chances of getting/keeping a job?	1	2	3	4	5
14	When you see the scar, do you remember the former traumatic event accurately?	1	2	3	4	5
15	Do you ever try to hide the scar?	1	2	3	4	5

Appendix B 

**Table 7:** Scale of basic activities of daily living (ADL) Each of the six items is ranked in three levels: autonomous = 0, partial assistance = 1, total assistance = 2, and an ADL score > 6 = a sign of dependence [23].

Activity	Description	Score
Hygiene	Autonomous	0
	Partial assistance for one part of the body	1
	Assistance for several parts of the body or toileting impossible	2
Dressing	Autonomous	0
	Dresses but needs assistance with shoes	1
	Needs assistance in choosing clothing, getting dressed and remains partially or completely undressed	2
Toileting	Autonomous	0
	Needs to be accompanied; needs assistance	1
	Does not go to the toilet; does not use the toilet or urinal	2
Locomotion	Autonomous	0
	Needs assistance	1
	Bedridden	2
Continence	Continent	0
	Occasional incontinence	1
	Permanent incontinence	2
Meals	Autonomous	0
	Needs assistance to cut meat or peel fruit	1
	Total assistance or artificial feeding	2
Total		
